# Malaria and Irrigated Crops, Accra, Ghana

**DOI:** 10.3201/eid1108.041095

**Published:** 2005-08

**Authors:** Eveline Klinkenberg, P.J. McCall, Ian M. Hastings, Michael D. Wilson, Felix P. Amerasinghe, Martin J. Donnelly

**Affiliations:** * Liverpool School of Tropical Medicine, Liverpool, United Kingdom;; † International Water Management Institute (West Africa), Accra, Ghana;; ‡ Noguchi Memorial Institute for Medical Research, Legon, Ghana;; § International Water Management Institute Headquarters, Colombo, Sri Lanka

**Keywords:** malaria, plasmodium, Urbanization, parasitaemia, anemia

## Abstract

We investigated the prevalence of malaria and associated risk factors in children living in urban Ghana. Malaria prevalence was associated with low hemoglobin concentration, low socioeconomic status, and higher age. Our findings indicate that African urban poor are seriously affected by malaria and that irrigated agriculture may increase this risk.

Malaria is predominantly a rural disease in Africa. Previous studies have shown that *Anopheles* mosquito breeding decreases with increasing proximity to the center of urban areas (1,2). Although the complex factors that contribute to malaria risk are not fully understood (2), availability of vector breeding sites is clearly essential. Urban agriculture, promoted as a means of increasing food security, improving nutrition, and alleviating poverty (3), can, especially when irrigated, create breeding habitats that could increase malaria transmission in cities. This potential risk was indicated by other authors (3–6), but only a limited number of studies have attempted to quantify the impact of urban agriculture on malaria transmission (4,7,8), and virtually all used only entomologic parameters (e.g., the entomologic inoculation rate, an estimate of the number of infected bites received per person per unit of time) in their analyses. Such measures are only proxies of actual malaria risk, and no studies have assessed the malaria parasite prevalence, a direct indicator of the impact of malaria, in communities with and without urban agriculture. By 2025, an estimated 700 million people will live in urban communities in Africa, which is approximately double the current urban population (9). With such rapid expansion, identification of the risk factors for urban malaria requires urgent attention (10).

## The Study

**Figure 1:**
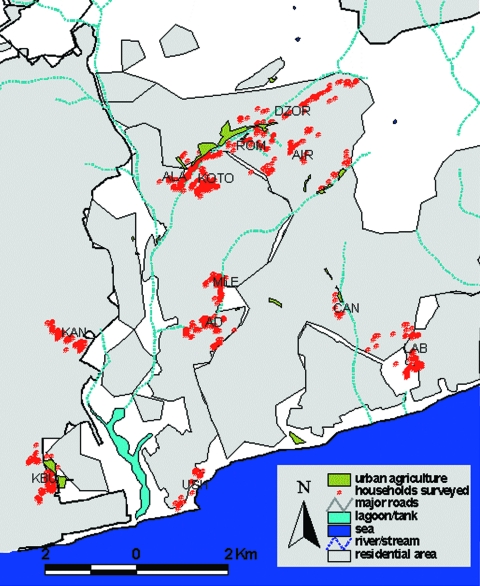
Location of urban agricultural (UA) sites and households surveyed within Accra, Ghana. Communities surveyed are shown with full name, UA or control (C), number of children sampled, and malaria prevalence. AIR, (Airport, UA, n = 77, 19.5%); ALA, (Alajo UA, n = 166, 15.1%); DZOR, (Dzorwulu UA, n = 132, 19.7%); KBU, (Korle Bu, UA, n = 181, 8.8%); KOTO, (Kotobabi, UA, n = 219, 18.3%); ROM, (Roman Ridge, UA, n = 105, 22.9%); CANT, (Cantonments, UA, n = 23, 13.0%); MLE, (Kokomlemle, C*, n = 160, 20.6%); AD, (Asylum Down, C*, n = 160, 11.3%); KAN, (Kaneshie, C, n = 159, 19.5%); LAB, (Labonie/LA, C, n = 175, 9.7%); USH, (Ushertown, C, n = 200, 6.5%). Communities marked C* were originally identified as control communities but small UA sites were later identified close to them.

**Figure 2:**
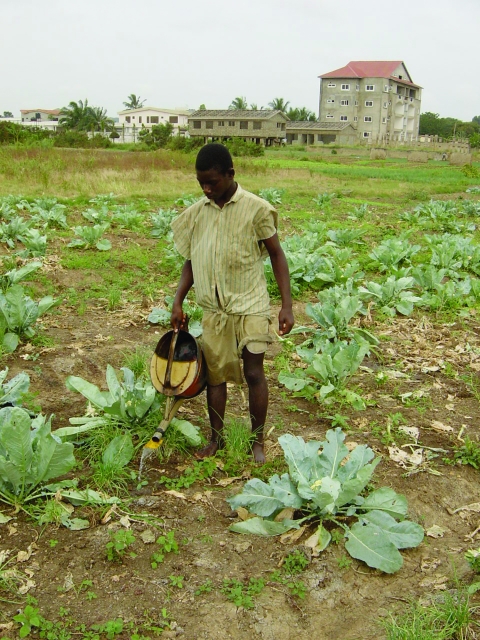
Commercial irrigated vegetable production in urban Accra, Ghana. Courtesy of Dr. Guy Barnish, Liverpool School of Tropical Medicine.

From October 2002 to January 2003, we investigated malaria parasite prevalence in central Accra, Ghana, in communities bordering irrigated urban agriculture areas and in control communities (defined as sites located >1 km from an urban agricultural area, based on the likely appetitive flight distance of female mosquitoes) (11) (Figure 1). Communities around the main agricultural sites in Accra were selected and based on them representative control communities in terms of socioeconomic status, housing and crowding were selected. Different types of urban agriculture exist: basic backyard farming in or around the house, cultivation of stable crops such as maize on (temporary) fallow land, and cultivation of ornamental plants, mostly along roadsides. An important part of agriculture in the city is commercial cultivation of vegetables, such as lettuce, onion, and cabbage (Figure 2). These crops are irrigated from wells or streams with watering cans, and crops are sometimes cultivated on raised beds with water-filled furrows. Irrigated farming has the greatest potential to create additional breeding sites, and irrigated, open-spaced vegetable farming has been linked to higher anopheline densities in Kumasi, Ghana (4). The study focused on this type of urban agriculture, which refers to irrigated, open-spaced, commercial vegetable production.

In the selected communities, we conducted a cross-sectional house-to-house survey to assess malaria parasitemia and hemoglobin (Hb) concentration in children 6 to 60 months of age. A team consisting of technicians and trained enumerators went house to house to collect data. Houses were selected arbitrarily and queried regarding the presence of children <5 years of age. For each community, the whole area was covered to account for spatial heterogeneity. If most compounds or houses had children <5 years of age, houses were omitted to obtain the target sample size of 150 children from the community.

Informed consent was obtained from each child's caregiver. Thick and thin blood films were collected and read according to standard World Health Organization protocols. Hb levels were assessed by using a blood hemoglobin photometer (HemoCue, Angelholm, Sweden). Children with parasitemias or Hb levels <8.0 g/dL were provided free treatment at a local clinic. The epidemiologic data were related to proximity to sites of urban agriculture, socioeconomic status based on household assets following a World Bank template (12), and possible confounding factors obtained by questionnaire from the child's caregiver. The location of each house, study site boundaries, landmarks, and urban agricultural areas were mapped by using a hand-held global positioning system. For each household in the urban agricultural communities, the shortest distance to the nearest agricultural site was calculated by using Arcinfo (ESRI, Redlands, CA, USA). Ethical approval was granted by the Liverpool School of Tropical Medicine and the University of Ghana, Legon.

A total of 1,757 children from 938 households in 12 different communities were enrolled in the study. Table 1 shows the baseline characteristics of the children with *Plasmodium*-positive and -negative slides, and Table 2 shows the characteristics for the urban agricultural and control communities. Of 261 infections detected, 258 were *P. falciparum*, 2 were *P. malariae*, and 1 was a mixed infection with *P. falciparum* and *P. malariae*. The average Hb level was 10.82 g/dL (SD 1.47), and 78 (4.5%) of 1,738 children had moderate-to-severe anemia (Hb <8.0 g/dL). Overall malaria parasite prevalence was 14.9% (261/1,757, range 6%–22%) and was higher in communities around urban agricultural sites than in control communities (16.5% and 11.4%, respectively, odds ratio [OR] 1.53, 95% confidence interval [CI] 1.10–2.14, p = 0.008). In a univariate analysis (Pearson chi square for binominal variables and *t* test for continuous variables), Hb concentration (negative association, p<0.001); moderate-to-severe anemia (OR 3.49, 95% CI 1.98–6.11, p<0.001); having netting in front of  windows, doors, or both (OR 0.65, 95% CI 0.46–0.92, p = 0.012); socioeconomic status (negative association, p<0.001); and age (positive association peaking at ≈3 years of age, p = 0.002) showed significant associations with presence of malaria parasites. Reported bed net use by a household was 33% (range 6%–53% in different communities) but was not significantly associated with presence of malaria parasites in the blood.

**Table 1:** Summary of variables measured for children with and without malaria parasites, with results of univariate (Pearson chi-square or t) tests*

Variables	*Plasmodium*-positive blood slide (n = 261)	*Plasmodium*-negative blood slide (n = 1,496)	p value
Mean Hb, g/dL (SD)	10.17 (1.62)	10.94 (1.42)	<0.001
Hb <8 g/dL, %	11.3 (29/257)	3.3 (49/1,481)	<0.001
Mean age, months (SD)	36.44 (16.03)	32.92 (17.19)	<0.001
Mean socioeconomic score† (SD)	1.42 (0.99)	1.74 (0.98)	<0·001
Male (%)	123 (47.1)	739 (49.4)	0.498
Travel to village‡ (%)	17 (6.5)	93 (6.2)	0.855
Taken malaria medication in last 2 wk§ (%)	63 (24.1)	344 (23.0)	0.686
History of fever§ (%)	64 (24.5)	293 (19.6)	0.067
HH with reporting bed net use (%)	89 (34.1)	499 (33.4)	0.814
HH who spray weekly¶ (%)	71 (27.2)	435 (29.1)	0.537
HH with netting at windows/doors (%)	208 (79.7)	1,282 (85.8)	0.012
HH without ceiling (%)	77 (29.8)	382 (25.6)	0.147

*Hb, hemoglobin; HH, household. †Composite measure of socioeconomic status used was the asset factor score of the World Bank for Ghana (http://www.worldbank.com/hnp). ‡Persons who had traveled to a rural (potentially malarious) area in the previous 3 weeks. §In the last 48 hours, as reported by the caregiver. ¶Proprietary brands of insecticide aerosols.

**Table 2:** Summary statistics for variables measured in children in communities near urban agricultural sites and control communities, with results of univariate (Pearson chi-square or t) tests*

Variables	Children in urban agricultural communities (n = 1,223)†	Children in control communities (n = 534)	p value
Children with *Plasmodium*-positive slide, %	16.4 (200/1,223)	11.4 (61/534)	0.008
Mean Hb, g/dL (SD)	10.93 (1.46)	10.59 (1.46)	<0.001
Hb <8 g/dL, %	3.4 (41/1,215)	5.5 (29/529)	0.039
Mean age, months (SD)	33.3 (17.1)	33.8 (17.0)	0.601
Mean socioeconomic score‡ (SD)	1.78 (0.96)	1.49 (1.02)	<0.001
Travel to village§, %	7.9	2.4	<0.001
Taken malaria medication in last 2 wk¶, %	23.5	22.3	0.600
History of fever¶#, %	21.2	18.2	0.155
HH reporting bed net use, %	37.7	24.2	<0.001

*Hb, hemoglobin; HH, household. Control communities were those >1 km from an urban agricultural area. †Number of children in the urban agricultural community group is higher because small plots of agriculture were discovered in 2 communities originally designated control sites. If these 2 communities were omitted from the analysis, similar results were obtained and significance remained the same except for children with moderate-to-severe anemia, which was no longer significant (p = 0.065) (data not shown). ‡Composite measure of socioeconomic status used was the asset factor score of the World Bank for Ghana (http://www.worldbank.com/hnp). §Persons who had traveled to a rural (potentially malarious) area in the previous 3 weeks. ¶As reported by the caregiver. #In the last 48 h.

A generalized linear mixed model (GLMM) approach, using an SAS macro (Glimmix 800, SAS Inc., Cary, NC, USA) that allowed a logistic link function, was used to investigate the association between putative predictor variables and malaria parasite prevalence. Covariates with p<0.1 in the univariate analysis were entered in the multivariate model. Household was nested within community, and both variables were treated as random effects. Age was divided into the following groups: 6–12, 13–24, 25–36, 37–48, and 49–60 months. Hb was entered as a continuous variable. Malaria parasitemia was significantly associated with Hb (negative association, p<0.001), age group (positive association, p<0001), and socioeconomic status (negative association, p = 0.0035). The effect of urban agriculture was marginally below significance (p = 0.0647), possibly because of reduced statistical power. Having netting in front of windows or doors was no longer significant (p = 0.3638), presumably because presence of nets was associated with a higher socioeconomic status (p<0.001).

In urban agricultural communities, GLMM analysis with parasitemia as the outcome was conducted with age group, distance to an urban agricultural site, socioeconomic status, and house effects. The Hb level was omitted because it was likely to be the result of malaria infection and its inclusion could obscure the effect of distance. Two of the districts, Mle (p = 0.021) and Kbu (p = 0.014), showed decreases in prevalence with distance from an urban agricultural site; the odds of infection were reduced ≈50% every 100 m from the site. However, these results need to be interpreted with caution because it is difficult to detect a putative decrease in prevalence with distance against the noise introduced by small, unidentified, often transitory, breeding sites. Their presence may explain why 2 sites, Rom (p = 0.043) and Dzor (p = 0.039), showed a significant increase in prevalence with distance, while 2 others, Air and Koto, showed a significant effect when distance in 100-m intervals was cross-tabulated with prevalence (p<0.001, Fisher exact test). Since unidentified breeding sites may also introduce unknown data structuring that cannot be incorporated into a GLMM, the probabilities obtained may be lower than are appropriate.

## Conclusions

The parasitemia levels obtained in this study are worrisome because high-density urban African populations are not often considered particularly vulnerable to malaria infection. In other West African urban areas, malaria prevalence rates from 2% to 16% have been reported with large variation between communities (5,13). Recently, several authors focused attention on urban malaria (2,12) and stressed the need to investigate risk factors for urban malaria. In our study, the parasitemic children were more likely to be anemic, have a lower socioeconomic status, and live in a community close to areas of urban agriculture. Since recent travel to a rural area did not affect outcome, local malaria transmission is indicated. Our entomologic studies in these study areas (unpub. data) have found *Anopheles gambiae* S form breeding in irrigation water at urban agricultural sites and resting at higher densities in houses in urban agricultural communities.

These findings are based on a point prevalence survey in the dry season. Although we continue to obtain data during the wet season, analysis of data indicates that the urban poor in Africa may be at higher risk for malaria than expected and that malaria can no longer be regarded only as a rural phenomenon. This finding is of great concern because in Africa the current urban population growth rate of 3.5% is >3 times the rural population growth rate, and by 2015 a total of 25 countries in sub-Saharan Africa will have urban populations larger than the rural populations (9). Although levels of transmission in urban areas may be lower than in contiguous rural areas, high population densities and possible lower immunity (6) may result in more disease impact in urban settings. Furthermore, although not the sole cause, irrigated urban agriculture may further increase the risk for malaria by providing suitable breeding sites. Further research on the interaction between type of urban agriculture and vector biology is needed because most African cities irrigate agricultural areas with water from polluted sources that is generally not favored by malaria vectors, although several studies have reported anophelines breeding in heavily contaminated water (14,15). The advantages of urban agriculture for alleviating poverty are numerous, but care must be taken that unregulated growth does not compromise its success. Integration of the activities of municipal authorities, agriculturalists, health professionals, and communities is essential to reduce the existing impact of malaria and to prevent future increases.
